# Exogenous SA Applications Alleviate Salinity Stress via Physiological and Biochemical changes in St John’s Wort Plants

**DOI:** 10.3390/plants12020310

**Published:** 2023-01-09

**Authors:** Eun-Hae Kwon, Arjun Adhikari, Muhammad Imran, Da-Sol Lee, Chung-Yeol Lee, Sang-Mo Kang, In-Jung Lee

**Affiliations:** 1 Department of Applied Biosciences, Kyungpook National University, Daegu 41566, Republic of Korea; 2 Department of Statictics Graduate School, Kyungpook National University, Daegu 41566, Republic of Korea

**Keywords:** salinity stress, endogenous melatonin, salicylic acid application, St. John’s wort, ASMT, SNAT

## Abstract

The plant St. John’s wort contains high levels of melatonin, an important biochemical that has both beneficial and adverse effects on stress. Therefore, a method for increasing melatonin levels in plants without adversely affecting their growth is economically important. In this study, we investigated the regulation of melatonin levels in St. John’s wort by exposing samples to salinity stress (150 mM) and salicylic acid (0.25 mM) to augment stress tolerance. The results indicated that salinity stress significantly reduced the plant chlorophyll content and damaged the photosystem, plant growth and development. Additionally, these were reconfirmed with biochemical indicators; the levels of abscisic acid (ABA) and proline were increased and the activities of antioxidants were reduced. However, a significant increase was found in melatonin content under salinity stress through upregulation in the relative expression of tryptophan decarboxylase (*TDC*), tryptamine 5-hydroxylase (*T5H*), serotonin N-acetyltransferase (*SNAT*), and N-acetylserotonin methyltransferase (*ASMT*). The salicylic acid (SA) treatment considerably improved their photosynthetic activity, the maximum photochemical quantum yield (133%), the potential activity of PSⅡ (294%), and the performance index of electron flux to the final PS I electron acceptors (2.4%). On the other hand, SA application reduced ABA levels (32%); enhanced the activity of antioxidant enzymes, such as superoxide dismutase (SOD) (15.4%) and 2,2-diphenyl-1-picrylhydrazyl (DPPH) (120%); and increased polyphenol (6.4%) and flavonoid (75.4%) levels in salinity-stressed St. John’s wort plants. Similarly, SA application under NaCl stress significantly modulated the melatonin content in terms of ion balance; the level of melatonin was reduced after SA application on salt-treated seedlings but noticeably higher than on only SA-treated and non-treated seedlings. Moreover, the proline content was reduced considerably and growth parameters, such as plant biomass, shoot length, and chlorophyll content, were enhanced following treatment of salinity-stressed St. John’s wort plants with salicylic acid. These findings demonstrate the beneficial impact of salt stress in terms of a cost-effective approach to extract melatonin in larger quantities from St. John’s wort. They also suggest the efficiency of salicylic acid in alleviating stress tolerance and promoting growth of St. John’s wort plants.

## 1. Introduction

Salinity stress is considered a major environmental threat to agricultural crops [1,2]. It causes various physiological disorders in plants, such as oxidative stress, ROS (reactive oxygen species) accumulation, chlorophyll degradation, ion imbalance, and ultimately cell death [3,4,5,6,7]. Several strategies have been recommended to cope with the adverse effects of salt stress, including the use of biostimulants, such as plant growth-promoting rhizobacteria and arbuscular mycorrhizal fungi; increased use of organic matter; application of lime; and use of different irrigation patterns [8,9,10,11]. In addition to these methods, treatment with phytohormones, such as abscisic acid, gibberellin, jasmonic acid, and salicylic acid, is widely reported to confer stress tolerance in several crops [12,13,14,15].

Despite the adverse effects of salt stress, it has some beneficial impacts. It has been demonstrated that salt stress positively affects essential oil content in some medicinal plants. The essential oil content of *Thymus vulgaris* [16], *Calendula officinalis* [17], *Trachyspermum ammi* [18], and *Rosmarinus officinalis* [19] was significantly enhanced by salt stress. Additionally, several studies have shown that salt stress increases the levels of secondary metabolites in medicinal plants, including tannins, saponins, terpenes, alkaloids, and phenols, which exert significant therapeutic effects [20,21,22]. This phenomenon mainly occurs because the synthesis of major secondary metabolites is controlled by the shikimic or mevalonate pathway, which is in turn controlled and modified by environmental stresses, such as increased salinity [20]. Considering that the major industrial goal of medicinal plant agriculture is to promote the synthesis of bioactive compounds and secondary metabolites [5], salinity may be an important tool for enhancing the antioxidant capacity of plants and the secondary metabolites of various medicinal plants under stress.

Salicylic acid (SA) induces systemic resistance and improves plant metabolism in several crops [23,24]. Improving physiological attributes, such as starch, sugar, protein, and proline content, as well as essential oil production and defense systems (catalase, peroxidase, and ascorbic peroxidase activities) by SA can alleviate salt toxicity in feverfew (*Tanacetum parthenium*) plants [25]. SA induces cross-signaling in plants and activates necessary defense systems, such as antioxidants and amino acids [26,27]. Antioxidants neutralize negative oxygen radicals and minimize lipid peroxidation. Amino acids regulate proteins that induce gene expression to strengthen the defense system. Proline regulates growth regulators, such as ABA (abscisic acid), which maintains the water potential and balances ion systems that enhance the overall plant defense system [28,29,30].

Melatonin (N-acetyl-5-methoxytryptamine) is a circadian and seasonal pacemaker in animals and a growth regulator in plants [31,32,33,34]. Melatonin is also produced by bacteria, fungi, and some invertebrates [35]. Plants containing phytomelatonin [36] are often consumed as health supplements to relieve depression and promote sleep. In particular, medicinal plants contain a significant amount of phytomelatonin and have been used as a herbal medicine for centuries [36,37]. Aromatic/medicinal plants contain higher levels of phytomelatonin (0.3 µg/g plant tissue) than edible plants (1–10 ng/g plant tissue) [38].

*Hypericum perforatum* L. (St. John’s wort) has been used as a medicinal plant for thousands of years in Europe [39] and is still used for treating mild to severe depression [40,41,42]. Because St. John’s wort is a special plant that accumulates high melatonin levels [43], it is important to assess its physiological activity and melatonin content under abiotic stress in the context of global climate change and environmental stress. Therefore, we attempted to increase the phytomelatonin content by increasing the melatonin content in St. John’s wort by salinity treatment of seedlings. Previous studies have described various damages to crops as a result of salinity stress, such as ion toxicity, ROS production, stomatal conductance reduction, and photosystem pigment reduction, which can eventually lead to death [44,45]. However, our study focused on the finding that salinity stress increases the melatonin content in plants, which functions as a stress regulator. Since promoting the synthesis of bioactive compounds and secondary metabolites in medicinal plants is important for agricultural and pharmaceutical industries, salinity stress can be an effective method to increase the content of medicinal components in crops.

In the present study, we had two specific objectives. First, our study focused on increasing the endogenous melatonin content of St. John’s wort with salinity treatment to enhance the efficacy of St. John’s wort as a medicinal crop. Second, we determined how exogenous SA application improves the physiological characteristics of salinity-stressed St. John’s wort by measuring changes in growth, photosynthesis, and amino acid content. We believe that our findings provide insight into various aspects of melatonin production and St. John’s wort growth in terms of medicinal and industrial uses.

## 2. Results

### 2.1. Effects of SA Concentration on the Chlorophyll Content of St. John’s Wort Leaves under Salt Stress

**Figure 1:**
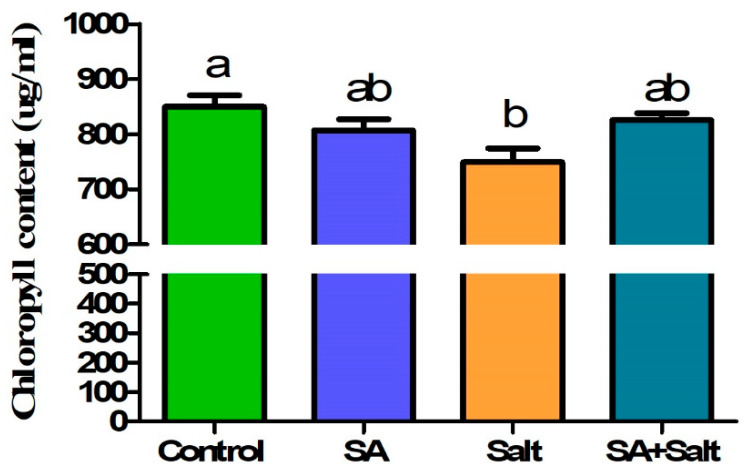
Leaf disk bioassay for selecting the appropriate concentration of salicylic acid for salinity-damaged St. John’s wort. Treatments: control, SA (0.25 mM salicylic acid), salt (150 mM NaCl), SA (0.25 mM salicylic acid) + salt (150 mM NaCl). Each value represents the mean ± SD (*n* = 20). Different letters on each bar represent statistically significant differences as evaluated by DMRT and *t*-test at *p* ≤ 0.05.

Compared with the control, salinity treatment resulted in a decrease in leaf chlorophyll content. However, salinity-stressed leaf disks treated with various concentrations of SA (0, 0.1, 0.25, and 1 mM) exhibited reduced damage from salinity stress compared with leaf disks exposed to salt stress alone (Figure 1). When salinity-stressed leaf disks were treated with SA at lower (0.1 mM) or higher (1 mM) concentrations, no remarkable changes were observed compared with only salinity-stressed leaf disks. However, compared with salinity-stressed leaf disks alone (Figure 1), SA treatment (0.25 mM) resulted in an increase in chlorophyll content (10.1%) (*p* ≤ 0.05). These results indicated that 0.25 mM of SA attenuated the suppressive effect of salinity stress on chlorophyll content. Therefore, selecting an optimal SA concentration (0.25 mM) is essential before applying it to seedlings.

### 2.2. Recovery Effect of Exogenous SA on St. John’s Wort Seedlings under Salinity Stress

#### 2.2.1. Plant Growth Characteristics and Chlorophyll Content

**Figure 2:**
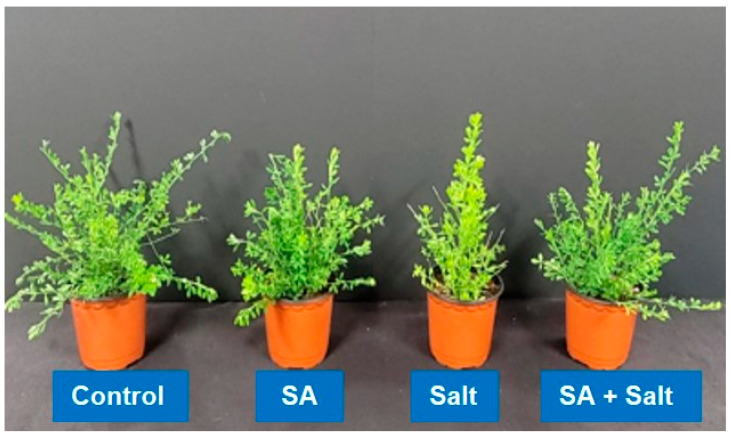
Morphological and physiological effect of salicylic acid on salinity-stressed St. John’s wort seedlings. St. John’s wort seedlings picture.

**Figure 3:**
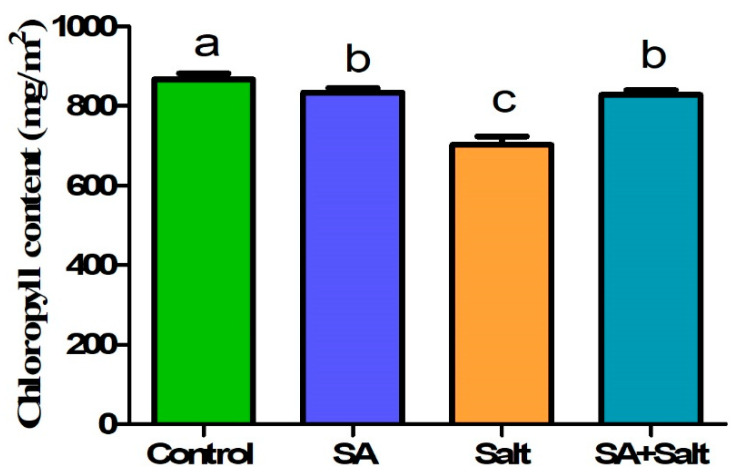
Impact of salicylic acid on chlorophyll content of St. John’s under normal and salinity stress conditions. Treatments: control, SA (0.25 mM salicylic acid), salt (150 mM NaCl), SA (0.25 mM salicylic acid) + salt (150 mM NaCl). Each value represents the mean ± SD (*n* = 10). Different letters on each bar represent statistically significant differences as evaluated by DMRT and *t*-test at *p* ≤ 0.05.

We evaluated the effect of salinity stress on St. John’s seedling growth using pot trials. Compared with unstressed seedlings, salinity negatively impacted growth parameters including fresh weight, plant height, number of leaves, and stem diameter (Table 1). However, in plants treated with SA under salinity stress, fresh weight, plant height, and stem diameter increased. Specifically, fresh weight, plant height, root length and stem diameter were improved by 35.11%, 24.8%, 34.21% and 52.23% in the salt in comparison with the corresponding heights of stressed plants (*p* ≤ 0.05). According to the results of this study, the application of SA to salinity-stressed and non-stressed plants substantially enhanced the physiological and morphological characteristics of the plants (Figure 2). Additionally, salinity-stressed plants (100 mM) showed reduced chlorophyll content. Compared with control plants, chlorophyll in salinity-stressed plants decreased significantly by 18.9% (*p* ≤ 0.05) (Figure 3); however, the exogenous application of SA (0.25 mM) alleviated stress in plants grown under saline conditions. A significant increase in chlorophyll content (17.8%) was observed when salinity-stressed plants were treated with 0.25 mM SA for 3 days (*p* ≤ 0.05) (Figure 3). This result indicates that root application of SA attenuated salinity-induced reduction in chlorophyll content. Additionally, as shown in Figure 2, we observed that St. John’s wort seedlings were severely damaged due to salinity stress; however, SA application reduced damage in salinity stressed plant.

Treatments: control, SA (0.25 mM salicylic acid), salt (150 mM NaCl), SA (0.25 mM salicylic acid) + salt (150 mM NaCl). Each value represents the mean ± SD (*n* = 10). In all data, error bars represent standard deviations and each data point represents the mean of ten replications. Different letters on each bar represent statistically significant differences as evaluated by DMRT and *t*-test at *p* ≤ 0.05

#### 2.2.2. Efficiency of the Photosystem (Fv/Fm, Fv/Fo, PI)

**Figure 4:**
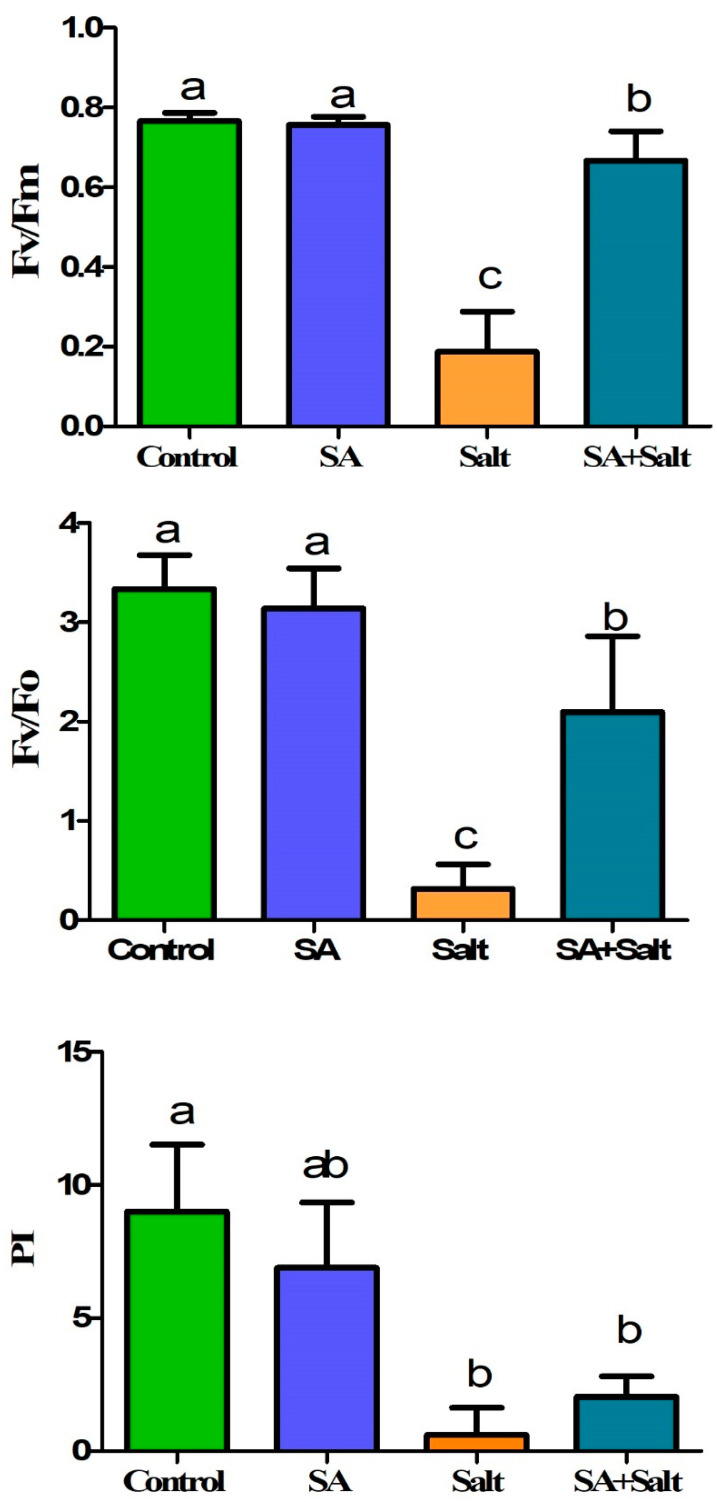
Effect of salicylic acid application on St. John’s wort photosynthetic parameters under salt stress “Fv/Fm; photosynthetic efficiency, Fv/Fo; potential activity of PSⅡ, PI; photosynthesis relative vitality”. Treatments: control, SA (0.25 mM salicylic acid), salt (150 mM NaCl), SA (0.25 mM salicylic acid) + salt (150 mM NaCl). Each value represents the mean ± SD (*n* = 10). Different letters on each bar represent statistically significant differences as evaluated by DMRT and *t*-test at *p* ≤ 0.05.

The reduction in photosynthetic parameters was observed under salinity treatment (100 mM). The maximum photochemical quantum yield (Fv/Fm), the potential activity of PSⅡ (Fv/Fo), and the performance index of electron flux to the final PS I electron acceptors (PI) decreased significantly in salinity-stressed plants by 63, 83, and 96% (*p* ≤ 0.05), respectively, compared with control plants (Figure 4). In contrast, exogenous salicylic acid (0.25 mM) alleviated the stress of plants grown in salty conditions. Notable enhancement in Fv/Fm (133%), Fv/Fo (294%), and PI (238%) was detected when the salinity-stressed seedlings were treated with 0.1 mM SA over 3 days (*p* ≤ 0.05). Over three days of SA treatment, SA application alleviated the decrease in photosynthetic parameters in stressed plants. This result indicated that salinity-induced reductions in photosynthetic parameters were attenuated by root application of SA.

#### 2.2.3. Influence of SA Application on Regulation of Endogenous ABA Content under Salinity Stress

**Figure 5:**
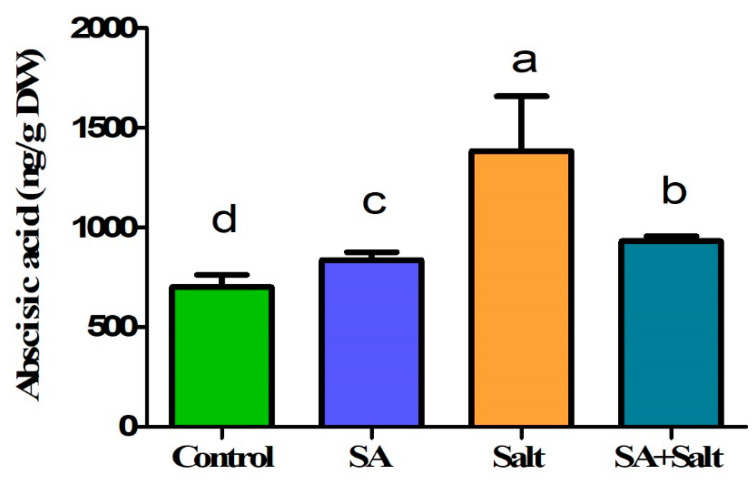
Effect of salicylic acid treatment on endo-ABA (abscisic acid) content of St. John’s wort under salt stress. Treatments: control, SA (0.25 mM salicylic acid), salt (150 mM NaCl), SA (0.25 mM salicylic acid) + salt (150 mM NaCl). Each value represents the mean ± SD (*n* = 3). Different letters on each bar represent statistically significant differences as evaluated by DMRT and *t*-test at *p* ≤ 0.05.

Endogenous ABA content was measured to determine the influence of 0.25 mM SA treatment on the recovery of salinity-stressed (100 mM) seedlings. Salinity-stressed seedlings exhibited a significantly higher ABA content than normal seedlings. In contrast, ABA content decreased significantly by 32.7% in salinity-stressed plants treated with 0.25 mM SA (Figure 5). Furthermore, ABA content was measured in plants under normal conditions, both with and without SA treatment. Seedlings treated with 0.25 mM SA showed a slight increase in ABA content by 19.5% when compared with the control (*p* ≤ 0.05). Overall, our findings showed that 0.25 mM SA treatment resulted in reduced ABA levels in 100 mM salinity-stressed seedlings.

#### 2.2.4. Estimation of Amino Acid Levels

Seventeen amino acids were measured at different concentrations in St. John’s wort seedlings (Table 2). Salinity stress increased proline and cysteine contents in salinity-stressed St. John’s wort seedlings compared with seedlings under normal conditions over 8 days. Proline (Pro) was markedly increased by 63% in salinity-stressed plants. Similarly, cystine (Cys) was markedly increased by 36% compared with the control (*p* ≤ 0.05). Moreover, the contents of all 17 amino acids decreased upon SA treatment in salinity-stressed plants. These findings indicate that 0.25 mM SA ameliorated amino acid content during salinity stress (100 mM).

#### 2.2.5. Determination of Enzymatic and Nonenzymatic Antioxidant Activities (DPPH, SOD, Phenol, and Flavonoid)

**Figure 6:**
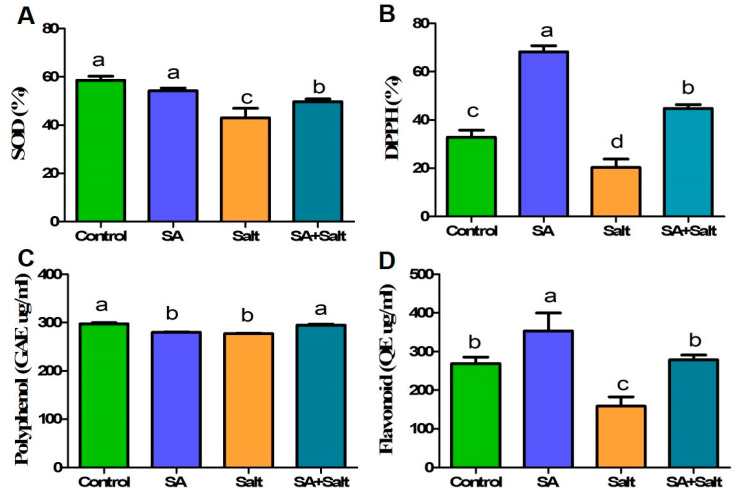
Determination of the enzymatic and non-enzymatic antioxidant activity of St. John’s wort under salt stress condition after salicylic acid treatment. Treatments: control, SA (0.25 mM salicylic acid), salt (150 mM NaCl), SA (0.25 mM salicylic acid) + salt (150 mM NaCl). Each value represents the mean ± SD (*n* = 3). Different letters on each bar represent statistically significant differences as evaluated by DMRT and *t*-test at *p* ≤ 0.05.

Compared with water alone, SA treatment enhanced antioxidant activity (except for polyphenol and SOD). DPPH and flavonoid contents increased by 108% and 31%, respectively, in St. John’s seedlings under normal conditions at the end of the experiment (8 days) (Figure 6). However, SA-treated plants had lower polyphenol and SOD contents on day 8 than the untreated controls (*p* ≤ 0.05). In addition, salinity stress led to a reduction in polyphenol (6.9%), DPPH (38.03%), SOD (26.45%), and flavonoid (41%) contents compared with those in untreated plants over 8 days (*p* ≤ 0.05). Compared with seedlings treated only with salinity, SA application to salinity-stressed seedlings increased polyphenol, DPPH, SOD, and flavonoid contents by 6.44%, 120%, 15.4%, and 75.4%, respectively, over the course of 8 days (*p* ≤ 0.05). SA-treated and untreated plants exposed to salinity showed notable differences in antioxidant activity and content.

### 2.3. Effects of SA Application on Regulation of Endogenous Melatonin and Transcriptional Response under Salinity Stress

**Figure 7:**
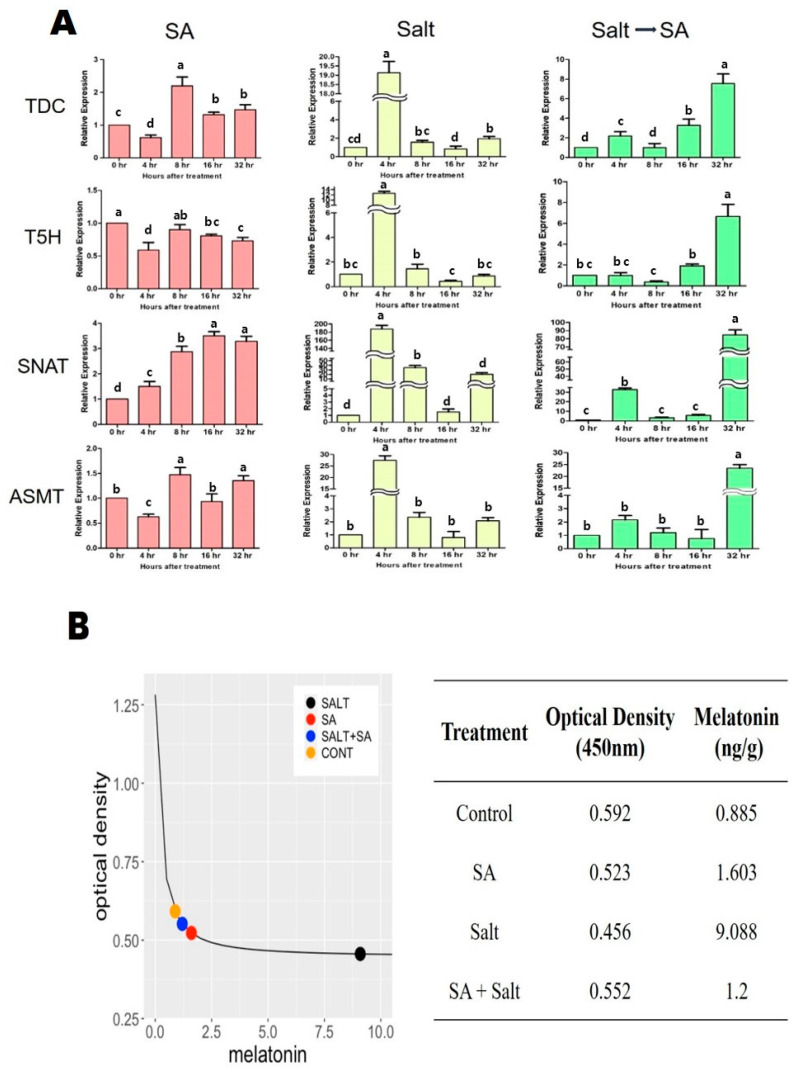
Impact of salicylic acid application on (**A**) endogenous melatonin content and (**B**) relative expression of genes (*TDC*, *T5H*, *SNAT* and *ASMT*) associated with melatonin synthesis in salinity-damaged St. John’s leaves. Treatments: control, SA (0.25 mM salicylic acid), salt (150 mM NaCl), SA (0.25 mM salicylic acid) + salt (150 mM NaCl). Each value represents the mean ± SD (*n* = 3). In all data, error bars represent standard deviations and each data point represents the mean of at least three replications. Different letters on each bar represent statistically significant differences as evaluated by DMRT and *t*-test at *p* ≤ 0.05.

The effects of salinity stress and SA treatment on endogenous melatonin content were measured in St. John’s wort seedlings, and were further validated through the relative gene expression associated with the melatonin biosynthesis pathway such as TDC, T5H, SNAT, and ASMT. When treated with salinity stress, the endogenous content of melatonin inside of the seedlings was found to increase by 926.9% compared to the non-stressed group (Figure 7A). Gene expressions (TDC, T5H, SNAT, and ASMT) in the melatonin biosynthesis pathway were increased by 1800%, 1160%, 18,640%, and 2640%, respectively, 4 h after salt treatment compared to the non-salinity stressed group, which explains the rapid increase in melatonin content in salinity-stressed St. John’s wort (Figure 7B). On the other hand, when SA treatment was performed on salt-stressed St. John’s wort, the melatonin content in the plants was rapidly reduced by 87% compared to salt-stressed seedlings (Figure 7A). Similar to the internal melatonin content, gene expressions were found to be very low compared to plants treated with salt stress (Figure 7B). Interestingly, while gene expressions in the melatonin synthesis pathway were drastically increased within 4 h after salinity stress, it was confirmed that the expressions gradually increased and showed the highest 32 h later after SA treatment on salinity-stressed seedlings (Figure 7B). Compared to control plants (non-stressed and non-treated), salinity-stressed plant treated with SA exhibited the increased content of endogenous melatonin by 36% and the upregulated expressions of *TDC*, *T5H*, *SNAT*, and *ASMT* (650%, 566%, 8360%, and 2246%) (Figure 7B). Overall, our findings indicated that application of SA in salinity-damaged seedlings decreased the endogenous melatonin content and *TDC*, *T5H*, *SNAT*, and *ASMT* expressions compared to untreated salinity-stressed plants but showed a higher amount of endogenous melatonin and melatonin synthesis gene expressions than control plants.

## 3. Discussion

Our research examined how salicylic acid (SA) impacts St. John’s wort under salinity stress and also studied how salinity treatment increases endogenous melatonin levels. SA treatment on salinity-damaged seedlings made some recovery in plant growth and development, as evidenced by improved growth parameters. In comparison to stressed plants in the absence of SA, plants receiving SA treatments (unstressed and stressed) maintained increased height, root length, weight, leaves, and stem diameter. Furthermore, we found that SA application enhanced the activities of antioxidants and reduced proline and ABA contents relative to the effects detected in untreated stressed plants. Probably, these functions are achieved through amelioration processes related to photosynthesis and other metabolic processes.

We measured the expression of several genes associated with the melatonin biosynthesis pathway in St. John’s wort following salinity and SA treatments. Melatonin biosynthesis begins with tryptophan and consists of four enzymatic steps; however, at least six enzymes are known to be involved—tryptophan decarboxylase (*TDC*), tryptophan hydroxylase, tryptamine 5-hydroxylase (*T5H*), serotonin N-acetyltransferase (*SNAT*), N-acetyl serotonin methyltransferase (*ASMT*), and caffeic acid O-methyltransferase (*COMT*) [46]. We evaluated the effect of salinity stress and exogenous SA application on the expression of *TDC*, *T5H*, *SNAT*, and *ASMT* in St. John’s wort seedlings. Salinity exposure resulted in a marked increase in the expression of *TDC*, *T5H*, *SNAT*, and *ASMT* compared with the other treatment groups. The highest expression was observed 4 h after salinity treatment, which was followed by a gradual decrease in expression. Previous studies demonstrated that melatonin self-regulates the expression of genes associated with biosynthesis and that *TDC*, *SNAT*, *ASMT*, and *COMT* are expressed under stress conditions, thus producing a drastic increase in the levels of endogenous melatonin [47]. Likewise, our findings indicated that salinity stress not only rapidly increased the expression of *TDC*, *SNAT*, *ASMT*, and *COMT* but also increased the endogenous melatonin content in plants. Our results are consistent with other studies in which salt treatment increased the expression of *ASMT*, *TDC*, *SNAT*, and *T5H* in peanut seedlings [48] and also induced the accumulation of melatonin in various plants [49,50,51]. These results indicate that melatonin may be involved in a mechanism underlying resistance to salt stress by rapidly increasing melatonin in a salinity stress environment.

Furthermore, our findings confirmed that SA treatment following salinity stress resulted in a decrease in the endogenous melatonin content and gene expression of *TDC*, *SNAT*, *ASMT*, and *COMT* compared to salinity-stressed seedlings but still showed an increase compared to control. Previous reports have shown that the exogenous application of SA at nontoxic concentrations to susceptible fruits and vegetables enhances resistance to biotic stresses [52,53]. Therefore, to ameliorate salinity stress, external SA was applied to salinity-stressed seedlings. We observed that salinity stress leads to a detrimental effect in plants and this was proved with growth parameters and physiological indicators (Figure 2, Figure 3, Figure 4, Figure 5 and Figure 6 and Table 1 and Table 2) despite higher melatonin accumulation. However, exogenous SA application at an appropriate concentration to salinity-stressed plants not only increased endogenous melatonin but also augmented stress tolerance in St. John’s wort, which was confirmed with enhanced physiological and biochemical factors (Figure 2, Figure 3, Figure 4, Figure 5 and Figure 6 and Table 1 and Table 2). Thus, our findings of the present study suggest that SA gradually increases melatonin content, thereby activating the plant’s immune system and resistance against salt stress. Considering not only the expression of these genes but also the growth and development from each treatment (Figure 7A,B), we conclude that SA is effective for recovering the damage to plants caused by salt treatment in St. John’s wort, which is consistent with that of previous studies [54,55,56,57,58,59]. 

ABA is involved in the maintenance of cell turgor pressure and the production of osmoprotectants and antioxidant enzymes, which are important messengers in adaptive mechanisms against abiotic stress in plants [60,61]. The present study shows that salinity treatment increased the endogenous ABA content and exogenous SA treatment in salinity-stressed plants decreased the ABA content compared with salinity-stressed plants. These results indicate that exogenous SA treatment mitigated salt-induced oxidative stress, which was confirmed by lower ABA levels in plants. Our results are consistent with those of previous studies that have shown that the application of exogenous SA ameliorates stress and decreases ABA levels in plants under abiotic stress [62,63]. We also observed that endogenous ABA levels in SA-treated plants were slightly increased compared with that in control plants, possibly as a result of the moderate stress caused by SA treatment. Previous results indicated that exogenous SA treatment at physiologically relevant concentrations caused moderate stress by generating H_2_O_2_, which in turn activated the antioxidative defense system including enzymatic and nonenzymatic antioxidants [64,65,66]. 

Salinity stress also affects photosynthetic components, such as enzymes, chlorophylls, and carotenoids. Changes in these parameters depend on the severity and duration of stress and on the plant species [67]. The decline in productivity observed in many plant species subjected to excess salinity is often associated with a reduction in photosynthetic capacity [68,69]. In the present study, we observed that photosynthetic efficiency and chlorophyll content were markedly decreased in salinity-stressed plants; however, exogenous SA application improved the efficiency of photosystem II and chlorophyll content. Several previous studies have demonstrated that SA effectively alleviates photosynthetic damage in Arabidopsis [70], tomato [71], and Caryophyllaceae [72]. Similarly, SA treatment increases the activity of certain enzymes, thereby stimulating chlorophyll biosynthesis or reducing chlorophyll degradation, leading to increased net photosynthesis [72,73,74], carbon fixation, stomatal conductance [75], and antioxidant activity [74] under salt stress tolerance. Consistently, our findings indicate that exogenous SA application generally contributes to the improved photosynthesis and the increased content of chlorophyll under salinity stress.

Because the production of ROS and the induction of oxidative stress by salinity are the main causes of reduced plant growth and productivity, regulation of ROS is an important process to avoid cellular cytotoxicity and oxidative damage [63]. To ameliorate the damaging effects of ROS, various defense mechanisms against salinity stress may be employed by plants, which predominantly involve enzymatic or nonenzymatic defense molecules [76,77,78]; however, a significant decrease in antioxidant enzyme activities was observed in soybean when the plants were treated with higher salinity concentrations [79]. In the present study, the activities of SOD and DPPH were reduced in salinity-stressed plants compared with the control plants. Similarly, flavonoid and polyphenol contents were also reduced in the salinity-stressed plants. Thus, oxidative stress in plants may occur through a decrease in antioxidant defenses, such as a decrease in the activities of main antioxidant enzymes. We observed that SA increased DPPH and flavonoid activities in both unstressed and stressed seedlings. Several studies have demonstrated that exogenous SA treatment enhances the activation of antioxidant enzymes. DPPH and flavonoids may neutralize negative oxygen radicals and relieve oxidative stress [80,81,82] and our results are consistent with these previous studies. Overall, our findings show that cross-talk between SA and antioxidants prevents senescence in plants.

In the present study, we also found that compared with the contents of other amino acids, the content of proline increased rapidly in stressed seedlings, which is consistent with the findings of previous studies on other plant species [83,84,85,86,87]. Proline functions as an osmoprotectant and ROS scavenger [88,89,90,91] and protects enzymes and membrane components that are sensitive to stress as a compatible solute [92,93]. Along with the maintenance of physiological mechanisms under stress, proline has been shown to improve the yield of various crops [94,95] and help plants withstand environmental challenges during the recovery period as a reservoir for organic nitrogen [26,96]. Furthermore, previous studies have found that proline content is reduced by SA treatment [97,98,99]. Similarly, in the present study, application of SA resulted in reduced proline content in stressed seedlings. Considering the ameliorating effect of SA on oxidative damage caused by salt stress, proline degradation may be triggered during stress relief [100,101] and is needed to maintain growth and development under salt stress [102]. Therefore, the results of our study and other reports indicate the stress-ameliorating effects of SA following salinity stress [98,103,104,105].

## 4. Materials and Methods

### 4.1. Experimental Design and Treatments

#### 4.1.1. Leaf Disk Bioassay

The effects of optimized salicylic acid concentrations on salt-stressed St. John’s wort were examined through foliar application in vitro. Leaf disks of St. John’s wort were sampled following growth under normal conditions. The leaf disks were placed in a Petri dish (60 × 15 mm) with filter paper (Wattman Co.) and treated with NaCl (100 mM, 150 mM, 200 mM, and 250 mM), salicylic acid (0.25 mM, 0.35 mM, and 0.45 mM), or sodium chloride+ salicylic acid with different concentrations. During the test, the leaf disks were sealed and kept at 25 °C for 3 days. Then, the chlorophyll content in these leaf disks was calculated according to the equation of Arnon (1949) [106]. Ground sprouts (100 mg) were extracted with 7 mL of dimethyl sulfoxide at room temperature for 24 h. The extraction mixture was filtered through a filter paper (Whatman No. 4) and a final volume of 10 mL of the extract was prepared in dimethyl sulfoxide. The absorbance of the extract was measured at 645 and 662 nm using a microplate spectrophotometer (Multiskan GO, Thermo Fischer Scientific, Vantaa, Finland).

$$\mathrm{Total}\;\mathrm{chlorophyll}\left(\mathrm{mg}/\mathrm{mL}\right)=20.2\times O.D._{645}+8.02\times O.D._{662}$$

where ‘OD’ is the optical density measured at the respective wavelengths.

#### 4.1.2. Plant Experiment 

St. John’s wort seedlings were provided by Heanam Duryusan Farm, Jeollanam-do, South Korea, and were transferred and cultivated under natural light in a greenhouse at Kyungpook National University, Daegu, South Korea (35.530° N, 128.360° E) with 25 °C/19 °C (day/night) temperature and 46.7% relative humidity on 1 May 2021. Seedlings were watered daily for 1 week to allow them to acclimatize to the new environment. Then, seedlings with uniform sizes were subjected to different treatments on 8 May 2021. The seedlings were divided into the following four groups: (i) control, grown with only distilled water (100 mL/plant); (ii) salt, irrigated with 150 mM NaCl (100 mL/plant) for the first 4 days and then only watered for 4 days; (ⅲ) SA, irrigated with 0.25 mM salicylic acid (100 mL/plant) for 4 days after watered for the first 4 days; and (ⅳ) SA + salt, irrigated with 150 mM NaCl (100 mL/plant) for the first 4 days. After 4 days, the plants were irrigated with 0.25 mM salicylic acid (100 mL/plant) for the last 4 days during total 8-day treatment period. Each treatment group consisted of 10 replicate plants. To prepare a salicylic acid solution (0.25 mM), 34.53 mg of the solute was dissolved in 1000 mL of distilled water and then diluted with distilled water. Samples were collected after all treatments finished for biochemical and molecular analysis. St. John’s wort samples were immediately frozen in liquid nitrogen, stored at −80 °C, and used for experiments after drying in a freeze dryer.

### 4.2. Biochemical Analysis of Plants 

#### 4.2.1. Chlorophyll Content and the Photosystem (Fv/Fm, Fv/Fo, PI)

The chlorophyll content in the leaves was determined using a portable CCM-300 Chlorophyll Content Meter (ADC BioScientific, Ltd., Herts, England) and the photosystem was measured with OS5p+ (Opti-Science, Inc, Hudson, NY, USA). 

#### 4.2.2. Quantitation of ABA Content

ABA content was determined using a previously described method [107,108]. Freeze-dried leaves of St. John’s wort (0.1 g) were ground and extracted with 10 mL of extraction solution. The extract was filtered, concentrated, dissolved in 5 mL of sodium hydroxide (1 N), and washed three times with dichloromethane (10 mL) to remove lipophilic material. pH of the aqueous phase was adjusted to 2.5 using hydrochloric acid (6 N HCl); then, ethyl acetate was added and the solution was mixed by vortexing. To remove phenolic compounds, the supernatant was evaporated to dryness and dissolved in phosphate buffer (pH 8.0). Polyvinylpolypyrrolidone was added to the extracted solution (phosphate buffer) and the solution was placed on a shaker for 40 min at 150 rpm. After the pH of the extract was adjusted to 2.5, ethyl acetate was added and vortexed. Then, the supernatant was collected and dried with a rotary evaporator. The dried residue was dissolved in diethyl ether. Finally, the extract was dried with nitrogen gas and methylated with diazomethane. The ABA content was quantified via GC–MS (Agilent 6890N Gas Chromatograph, Santa Clara, CA, USA). The response to ions was determined using a software package (ThermoQuset, Manchester, UK) [m/e of 162 and 190 for Me-ABA and 166 and 194 for Me-(2H6)-ABA].

#### 4.2.3. Measurement of Amino Acid Content

The amino acid content was quantified according to the method described by Waqas (2015) [109]. The freeze-dried leaf samples were hydrolyzed in 6 N HCl (1 mL) for 24 h at 110 °C and the extraction was concentrated and dried under vacuum for 24 h at 80 °C. The residue was then diluted with deionized water (2 mL) and evaporated; this procedure was repeated twice. Finally, the concentrated residue was dissolved in 0.02 N HCl (1 mL) and passed through a 0.45-µm filter. The solution was analyzed using a Hitachi L-8900 amino acid analyzer (Hitachi High-Technologies Corporation, Tokyo, Japan) with three replicates for each treatment.

#### 4.2.4. Determination of Enzymatic and Nonenzymatic Antioxidant Activities

Fresh leaf samples (0.1 g) were homogenized in 1 mL of 50 mM phosphate buffer (pH 7.0, 1 mM EDTA, 1% PVP), incubated at 4 °C for 10 min, and the mixture was centrifuged at 10,000 rpm for 10 min at 4 °C. The supernatant was used to determine superoxide dismutase (SOD) activity, which was measured using a SOD Assay Kit-WST (Dojindo Co., Ltd., Kumamoto, Japan). Based on the method of [110], DPPH radical scavenging activity as well as flavonoid, SOD, and polyphenolic contents were determined. The activity and absorbance of the mixture were measured using a MultiskanTM GO UV/Vis microplate spectrophotometer (Thermo Fisher Scientific, Waltham, MA, USA).

#### 4.2.5. Quantification of Melatonin Content

Melatonin content was analyzed using a Melatonin ELISA Kit (Enzo Life Sciences, Inc., Farmingdale, NY, USA). Quantification of melatonin was performed based on the manufacturer’s instructions. A freeze-dried sample was ground to a fine powder and approximately 0.1 g of it was homogenized with 1× stabilizer and cold ethyl acetate (125 µL and 750 µL) and then vortexed. The organic layer was removed by centrifuging at 1000× *g* for 3 min and then dried with nitrogen gas. Pellets were suspended and assayed in 125 µL of 1× stabilizer with at least two dilutions. The pretreated samples were placed in the appropriate well (Goat anti-Mouse IgG Microtiter Plate, one plate of 96 wells). After that, 50 µL of 1× melatonin tracer and 50 µL of 1× melatonin antibody was added to the wells in order. The plate was then sealed with a plate sealer and incubated at room temperature on a shaker for 1 h at 200 rpm. Then, the contents of the wells were emptied and washed three times using 400 µL of wash solution. When the final wash was complete, the contents were emptied and the wells were treated with 200 µL of the melatonin conjugate solution, sealed, and placed on a shaker at 300 rpm for 30 min. The wells were then washed with wash solution and 200 µL of TMB substrate solution was added to each well. After the samples were placed on a shaker for 30 min at 300 rpm, 50 µL of stop solution was added to each well. Finally, the optical density of the contents was measured using a MultiskanTM GO UV/Vis microplate spectrophotometer (Thermo Fisher Scientific, Waltham, MA, USA) at 450 nm.

#### 4.2.6. cDNA Synthesis and Real-Time PCR Analysis of Enzymes of the Melatonin Pathway

Total RNA was extracted from the leaves collected 4, 8, 16, and 32 h after exposing samples to different treatments. Total RNA was extracted with Trizol TRI Reagent Solution (ThermoScientific, Seoul, Republic of Korea) according to the manufacturer’s instructions. The obtained RNA was subjected to cDNA synthesis and quantitative PCR (qPCR) according to the detailed methods of Kazerooni (2018) [111] using a reverse transcription kit (BioFactTM RT, Daejeon, Republic of Korea). Two-step real-time polymerase chain reactions were conducted using an EcoTM real-time PCR system (Illumina) and real-time PCR master mix, which included SYBR Green I (Biofact) and 10 pmol of each primer. ECO Study (Illumina) was used to analyze gene expression data in triplicate. Relative expression levels of various genes (*TDC*, *T5H*, *SNAT*, and *OMT*) involved in the melatonin pathway were determined in control and treated plants (Supplementary Table S1).

### 4.3. Statistical Analysis

The experiments were conducted three times. The results were analyzed through one-way ANOVA using SAS On Demand and GraphPrism 5. A least significant difference test (*p* ≤ 0.05) was used to determine significant differences among treatments. Microsoft Excel 2017 was used to estimate the mean and standard deviation. Graphs were prepared using GraphPad Prism software (version 6.01; San Diego, CA, USA).

## 5. Conclusions

In conclusion, salinity enhances melatonin content in St. John’s wort seedlings; however, it has an adverse effect on plant growth. SA treatment of salinity-stressed St. John’s wort seedlings improved growth by modulating phytohormones and increasing antioxidant and amino acid levels. Based on this approach, we can extract melatonin in large quantities through salt stress treatment and also enhance the growth of plants for other medicinal purposes through SA application.

## Figures and Tables

**Table 1:** Growth parameters of salicylic acid on salinity-stressed St. John’s wort seedlings. St. John’s wort seedlings picture.

Treatment	Plant Height (cm)	Root (cm)	Total Plant Fresh Weight (g)	Stem Diameter (cm)	No. Leaf
Control	10.5 ± a	5.2 ± ab	8.3 ± a	0.37 ± a	193 ± a
SA	10.6 ± a	5.7 ± a	8.7 ± a	0.35 ± ab	194 ± a
Salt	6.6 ± c	3.5 ± c	4.8 ± c	0.2 ± c	92 ± b
SA + Salt	8.2 ± b	4.7 ± b	6.5 ± b	0.31 ± b	135 ± c

Treatments: control, SA (0.25 mM salicylic acid), salt (150 mM NaCl), SA (0.25 mM salicylic acid) + salt (150 mM NaCl). Each value represents the mean ± SD (*n* = 10). In all data, error bars represent standard deviations and each data point represents the mean of ten replications. Different letters on each bar represent statistically significant differences as evaluated by DMRT and *t*-test at *p* ≤ 0.05.

**Table 2:** Effect of salicylic acid on endo-amino acid content in salinity-stressed St. John’s wort seedlings.

Amino Acid	Treatment			
mg/g	Control	SA	Salt	Salt → SA
Asp	3.73 ± 0.0 a	3.24 ± 0.0 b	3.73 ± 0.0 a	3.15 ± 0.0 b
Thr	1.93 ± 0.0 b	1.67 ± 0.0 a	1.89 ± 0.0 b	1.64 ± 0.0 c
Ser	1.8 ± 0.0 a	1.58 ± 0.0 b	1.87 ± 0.0 a	1.64 ± 0.0 b
Glu	6.83 ± 0.0 a	6.16 ± 0.0 b	6.25 ± 0.0 a	5.51 ± 0.0 b
Gly	2.02 ± 0.0 a	1.74 ± 0.0 bc	1.83 ± 0.0 b	1.72 ± 0.0 c
Ala	2.43 ± 0.0 a	2.11 ± 0.0 b	2.55 ± 0.0 a	2.05 ± 0.0 b
Cys	0.14 ± 0.0 a	0.13 ± 0.0 a	0.19 ± 0.0 a	0.15 ± 0.0 a
Val	2.17 ± 0.0 a	1.89 ± 0.0 b	2.12 ± 0.0 a	1.82 ± 0.0 c
Met	0.0 8 ± 0.0 a	0.0 6 ± 0.0 a	0.0 7 ± 0.0 a	0.0 7 ± 0.0 a
Ile	1.63 ± 0.0 a	1.42 ± 0.0 c	1.56 ± 0.0 b	1.34 ± 0.0 d
Leu	3.43 ± 0.0 a	2.96 ± 0.0 c	3.18 ± 0.0 b	2.81 ± 0.0 c
Tyr	1.04 ± 0.0 a	0.86 ± 0.0 c	1.05 ± 0.0 ab	0.92 ± 0.0 bc
Phe	2.07 ± 0.0 a	1.81 ± 0.0 b	1.92 ± 0.0 b	1.74 ± 0.0 c
Lys	3.05 ± 0.0 a	2.61 ± 0.0 c	2.69 ± 0.0 b	2.6 ± 0.0 c
His	1.11 ± 0.0 a	0.96 ± 0.0 b	1.16 ± 0.0 a	0.91 ± 0.0 b
Arg	6.57 ± 0.0 a	6.07 ± 0.0 b	5.31 ± 0.0 c	4.71 ± 0.0 d
Pro	3.2 ± 0.0 a	2.87 ± 0.0 d	5.21 ± 0.0 a	2.95 ± 0.0 c

Treatments: control, SA (0.25 mM salicylic acid), salt (150 mM NaCl), SA (0.25 mM salicylic acid) + salt (150 mM NaCl). Each value represents the mean ± SD (*n* = 3). In all data, error bars represent standard deviations and each data point represents the mean of ten replications. Different letters on each bar represent statistically significant differences as evaluated by DMRT and *t*-test at *p* ≤ 0.05.

## Data Availability

Not applicable.

## Supplementary Materials

The following supporting information can be downloaded at: https://www.mdpi.com/article/10.3390/plants12020310/s1, Table S1: Details of genes: names, descriptions and oligonucleotide sequences used for qRT-PCR.
