# Supplementary material for: Exogenous SA Applications Alleviate Salinity Stress via Physiological and Biochemical changes in St John’s Wort Plants
Source: Plants (Basel). 2023 Jan 9;12(2):310. doi: 10.3390/plants12020310 (PMC9861905; doi:10.3390/plants12020310)
Supplement: Supplementary file 1 [file plants-12-00310-s001.zip › plants-2114926-supplementary.pdf]

Exogenous SA applications alleviate salinity stress via physiologic and biochemical regulation in St John's wort plants

Eun-Hae Kwon[1], Arjun Adhikari [1], Muhammad Imran[1], Da-Sol Lee[1], Chung-Yeol Lee[2], Sang-Mo Kang [1] and In-Jung Lee [1,*]

Table S1: Details of genes; name, description and oligonucleotide sequences used for qRT-PCR.

Gene Name	Description	Primer Sequence (5' to 3')
TDC	Tryptophan decarboxylase	L:GGCTCGGATGGGTCATCGTG<br>R:TCCGCAGCAGTCGTGGTAAC
T5H	Tryptamine 5-hydroxylase	L:CCTTCCTTGGCCGGAACCTC<br>R:TCTCGACGAGGAGGAGAGGGA
SNAT	Serotonin *N*-acetyltransferase	L:TAGACTCCGAGGCCGTCACC<br>R:TAGACTCCGAGGCCGTCACC
ASMT	*N*-acetylserotonin methyltransferase	L:GGTGACTCGTGGTGGCGAAA<br>R:AGGCTCCTCTCCCAACTGCT
